# Id2 promotes the invasive growth of MCF-7 and SKOV-3 cells by a novel mechanism independent of dimerization to basic helix-loop-helix factors

**DOI:** 10.1186/1471-2407-9-75

**Published:** 2009-03-04

**Authors:** Yuanguang Meng, Chenglei Gu, Zhiqiang Wu, Yali Zhao, Yiling Si, Xiaobing Fu, Weidong Han

**Affiliations:** 1 Department of Molecular Biology, Institute of Basic Medicine, Chinese PLA General Hospital, 28 Fu Xing Road, Beijing, 100853, PR China; 2 Department of Obstetrics and Gynecology, Chinese PLA General Hospital, 28 Fu Xing Road, Beijing, 100853, PR China

## Abstract

**Background:**

Inhibitor of differentiation 2 (*Id2*) is a critical factor for cell proliferation and differentiation in normal vertebrate development. Most of the biological function of Id2 has been ascribed to its helix-loop-helix motif. Overexpression of Id2 is frequently observed in various human tumors, but its role for invasion potential in tumor cells is dispute. We aimed to reveal the role of Id2 in invasion potential in poorly invasive and estrogen receptor α (ERα)-positive MCF-7 and SKOV-3 cancer cells.

**Methods:**

MCF-7 and SKOV-3 cells were stably transfected with the wild-type, degradation-resistant full-length or helix-loop-helix (HLH)-deleted Id2, respectively. Protein levels of Id2 and its mutants and E-cadherin were determined by western blot analysis and mRNA levels of Id2 and its mutants were determined by RT-PCR. The effects of Id2 and its mutants on cell proliferation were determined by [^3^H]-thymidine incorporation assay and the 3- [4, 5-dimethylthiazol-2-yl]-2,5-diphenyl tetrazolium bromide (MTT) dye method. The *in vitro *invasion potential of cells was evaluated by Transwell assay. Cell motility was assessed by scratch wound assay. The promoter activity of *E-cadherin *was determined by cotransfection and luciferase assays.

**Results:**

Ectopic transfection of the wild-type Id2 markedly increased the protein and mRNA expression of *Id2 *in MCF-7 and SKOV-3 cells; the protein level but not mRNA level was further increased by transfection with the degradation-resistant Id2 form. The ectopic expression of Id2 or its mutants did not alter proliferation of either MCF-7 or SKOV-3 cells. Transfection of the wild-type Id2 significantly induced the invasion potential and migratory capacity of cells, which was further augmented by transfection with the degradation-resistant full-length or HLH-deleted Id2. E-cadherin protein expression and transactivation of the proximal E-cadherin promoter were markedly suppressed by the degradation-resistant full-length or HLH-deleted Id2 but not wild-type Id2. Ectopic expression of E-cadherin in MCF-7 and SKOV-3 cells only partially blunted the invasion potential induced by the degradation-resistant HLH-deleted Id2.

**Conclusion:**

Overexpression of Id2 in ERα-positive epithelial tumor cells indeed increases the cells' invasive potential through a novel mechanism independent of dimerization to basic helix-loop-helix factors. E-cadherin contributes only in part to Id2-induced cell invasion when Id2 is accumulated to a higher level in some specific cell types.

## Background

Tumor metastasis is the highest cause of death in cancer patients. In carcinomas, the metastasis is thought to be a complex biological process. The first crucial step is the movement of cancer cells into tissue surrounding the tumor and vasculature [1]. During this step, a small proportion of epithelial tumor cells lose cell-cell adhesion and gain higher mobility, thus allowing them to invade the adjacent tissues [2,3]. Hence, the molecular events that contribute to the increased motility of tumor cells has become important for understanding tumor metastasis as well as for targets for potential therapeutic intervention in human cancers. Both human breast cancer MCF-7 and human ovarian carcinoma SKOV-3 cells are estrogen-receptor α (ERα)-positive epithelial tumor cell lines. They are usually used as experimental cell models because of their poorly invasive capacity [4,5]. These cell models are helpful for exploring genes aberrantly expressed in tumor cells that contribute to tumor metastasis [6-8].

Inhibitor of differentiation 2 (Id2) is one of the four members of the Id protein family [9]. In normal organisms, Id proteins are key regulators in development. They control lineage determination, differentiation, and proliferation in a variety of diverse cell types by regulating transcriptional networks [9-11]. Id mRNA and protein levels are elevated in diverse human tumor types [12-14]. By fueling several key features of tumor progression, including deregulated proliferation, invasiveness and metastasis, Id proteins contribute to multiple steps of tumorigenesis [13,15]. Although the general role of Id2 proteins has been considered pro-growth and anti-differentiation in various human tumors [13-15], their role in modulating invasion and metastasis of some specific tumor cells remain to be investigated.

All members of the Id protein family share a similar structure consisting of a highly conserved helix-loop-helix (HLH) domain [11]. Apart from the HLH structure, both the NH_2 _and COOH region sequences vary greatly among Ids [16]. The growing interest in the biology of Id proteins during the past decades has not substantially modified the primary function of "inhibitor of DNA binding" as originally assigned in 1990 [17]. This function has been ascribed to the HLH sequence motif, which mediates heterdimerization with the basic HLH (bHLH) transcription factors, ETS and PAX-DNA binding proteins, and retinoblastoma (Rb) tumor suppressor protein [17-20]. When engaged by Id proteins, the transcription factor is no longer able to bind to DNA target sequences and activate transcription. Although the HLH motif of Id2 lies at the center of a molecular network controlled by Id2, evidence suggests that Id2 performs its HLH-independent function prominently in some specific cell types. For example, in both interleukin-3 (IL-3)-dependent 32D.3 myeloid progenitors and U2OS osteosarcoma cells, the non-HLH region of Id2 performed a prominent apoptosis-promoting function [21].

Id2 protein is very unstable in cells. It contains a canonical D-box motif (RxxLxxxN) at residues 100 to 107. Recently, expression of Id2 with D-box mutation was found to be resistant to APC/C^Cdh1^-mediated degradation in cells, subsequently extending the half-life of Id2 to more than 10-fold that of the wild-type Id2 [22]. Thus, ectopic expression of the degradation-resistant form of Id2 protein in certain tumor cell types might provide beneficial effects for displaying Id2-mediated signaling and phenotypic traits, which might easily be overlooked with transfection of wild-type Id2.

We aimed to reveal the role of Id2 and its degradation-resistant mutants in cell invasiveness and migration in poorly invasive MCF-7 and SKOV-3 cancer cells positive for ERα. We were particularly interested in whether the HLH feature of Id2 was implicated in the Id2 function and the Id2 protein effect on the metastasis suppressor E-cadherin. Ectopic expression of the wild-type human Id2 markedly increased the *in vitro *invasion capacity of MCF-7 and SKOV-3 cancer cells, which was further augmented by transfection with the degradation-resistant form of Id2. Strikingly, the HLH-deleted Id2 form could also significantly increase cell invasiveness and migration, which indicates that the invasion-promoting role of Id2 is independent of its ability to dimerize with bHLH members. The invasion potential induced by Id2 was only partially associated with the down-regulation of the metastasis suppressor E-cadherin, suggesting that multiple molecules associated with tumor metastasis may be implicated in this process.

## Methods

### Plasmid constructs

The full-length coding region of the wild-type human Id2 in plasmid pLXSN [23], was obtained from Prof. Desprez (California Pacific Medical Center, San Francisco, CA). The plasmid pcDNA3-Id2-DBM containing the complete coding sequence of the full-length human Id2 with D-box mutation was kindly donated by Prof. Iavarone (Columbia University Medical Center, New York) [22]. The luciferase reporter gene construct (E-cadK1-*Luc*) containing the *E-cadherin *sequence and its mutants (E-cadK1-Luc-E-boxA, E-cadK1-Luc-E-boxB and E-cadK1-Luc-E-boxC) were provided by Dr. Eric R Fearon (University of Michigan Medical School, Ann Arbor, MI). The E-cadherin expression vector was described previously [8]. The Id2-DBM-δHLH fragment, lacking the entire HLH domain (codons 35 to 76 aa), was derived from pcDNA3-Id2-DBM by a sequential PCR scheme. In PCR reaction A, pcDNA3-Id2-DBM was amplified with 5'-atgaaagccttcagtcccgt-3' and 5'-gtccagggcgatcaggctcatcgggtcg-3' as the upstream and downstream primers, respectively, to give a 117-bp fragment. In PCR reaction B, pcDNA3-Id2-DBM was amplified with 5'-ccgatgagcctgatcgccctggactcgc-3' and 5'-ttagccacacagtgctttgc-3' as the upstream and downstream primers, respectively, to give a 189-bp fragment. Aliquots of each PCR product were purified after agarose gel electrophoresis and combined to serve as a template for a final PCR with the use of the PCR A upstream primer and the PCR B downstream primer. The 282-bp PCR product was then subcloned into *Hind *III and *BamH *I sites of pcDNA3.1 to generate pcDNA3.1-Id2-DBM-δHLH. The full-length Id2 cDNA derived from pLXSN-Id2 was subcloned into pcDNA3.1 at *BamH *I and *Xho *I sites to generate pcDNA3.1-Id2. The Id2-DBM fragment from pcDNA3-Id2-DBM was amplified by PCR and subcloned into pcDNA3.1 at *BamH *I and *Xho *I sites to generate pcDNA3.1-Id2-DBM. All constructs were confirmed by restriction enzyme mapping and DNA sequencing.

### Cell culture

The human breast cancer cell line MCF-7 and ovarian cancer cell line SKOV-3 were obtained from the American Type Culture Collection (ATCC, Rockville, MD). MCF-7 cells were maintained as monolayer cultures in Dulbecco's modified Eagle's medium (DMEM; Gibco, New York) supplemented with 10% fetal bovine serum (FBS; Hyclone, UT), 100 U/ml penicillin G and 100 μg/ml streptomycin. SKOV3 cells were maintained in RPMI1640 (Gibco) containing 10% FBS, 100 U/ml penicillin G and 100 μg/ml streptomycin in a humid atmosphere with 5% CO_2 _at 37°C.

### Cell transfection and small interfering RNA (siRNA)

MCF-7 and SKOV3 cells were seeded in 60-mm culture dishes before transfection. When the cell confluence reached 40–60%, cells were stably transfected with 5 μg pcDNA3.1-Id2, pcDNA3.1-Id2-DBM or pcDNA3.1-Id2-DBM-δHLH by use of the Superfect transfection reagent (Qiagen, Hilden, Germany), according to the manufacturer's instructions. The empty vector was used as a negative control. Two days post-transfection, MCF-7 cells were treated with 1000 μg/ml G418 (Gibco) and SKOV3 cells with 1 mg/ml G418 for 10–14 d and then were continuously cultured with 400 μg/ml G418.

For siRNA transfection, MCF-7 cells stably expressing Id2-DBM were seeded in 60-mm culture dishes and grown to 80% confluence. An amount of 4 μg siRNA duplexes were transiently cotransfected with use of Lipofectamine 2000 according to the manufacturer's recommendations (Invitrogen, Carlsbad, CA). Oligonucleotides for siRNA were chemically synthesized by Shanghai GeneChem Co. (Shanghai, China). The human *Id2*-specific siRNA, 5'-cacggatatcagcatcctg-3' (sense strand), corresponds to 504 to 522 bp (accession no. NM_002166) [24]. The unrelated siRNA sequence (sense strand, 5'-ttctccgaacgtgcacgt-3') was used as a control.

### Western blot analysis

The expression of Id2, E-cadherin and β-actin proteins was examined by western blot analysis as described previously [8]. Antibodies were monoclonal rabbit anti-Id2 antibody (1:500, Invitrogen), polyclonal rabbit anti-E-cadherin antibody (1:300, Santa Cruz Biotechnology), polyclonal Flag antibody (1:500, Sigma) or polyclonal rabbit anti-β-actin antibody (1:500, Santa Cruz Biotechnology). Blots were probed with the primary antibodies, washed and then incubated with horseradish peroxidase-labeled secondary antibodies (Santa Cruz Biotechnology), and binding was detected using enhanced chemiluminesence.

### RT-PCR

Total RNAs were extracted by the acid guanidium thiocyanate-phenol chloroform method with TriBlue reagent purchased from Tiangen Bio. (Beijing). cDNA was prepared with Superscript III RNase H^- ^reverse transcriptase (Invitrogen) and 2–5 μg of total RNA. The PCR primer sets 5'-atgaaagccttcagtcccgt-3' (sense) and, 5'-ttagccacagtgctttgc-3' (antisense) were used for amplifying Id2 and its derivatives. GAPDH was used as internal control with the following primer sets: forward primer, 5'-aaggtcggagtcaacggatt-3', and reverse primer, 5'-catgagtccttcacgatac-3'. The PCR products were fractionated by electrophoresis on a 1.5% agarose gel containing 0.5% ethidium bromide.

### Cell proliferation assays

Assay fro [^3^H]-thymidine incorporation (Yahui, Co. Beijing) and the 3- [4, 5-dimethylthiazol-2-yl]-2,5-diphenyl tetrazolium bromide (MTT) dye method were used to assess cell proliferation. For thymidine incorporation, cells in logarithmic growth phase were detached by use of 0.25% trypsin and seeded in 24-well plates in culture media with 10% FBS (1 × 10^4^/well). Media was changed every 24 h during the experiment. [^3^H]-thymidine (2 μCi) was added to fresh medium and incubated with cells for 1 h at 37°C. Cells were then washed, methanol fixed, and solubilized prior to scintillation counting. For MTT assays, 3 × 10^3 ^viable cells were plated in 96-well plates with culture medium containing 10% FBS. After incubation for 12 h, the medium was replaced with fresh medium containing 1% FBS and 400 μg/ml G418 for 6 or 8 days. Media was changed every 24 h during the experiment. MTT labeling reagent was added to fresh medium and incubated cells for 4 h at room temperature. Absorbance was examined at 590 nm by use of a microplate reader.

### Transwell assay (Boyden chamber invasion assay)

Invasion assays were carried out in modified Boyden chambers with 8-μm pore filter inserts for 24-well plates (BD Transduction) as described [8]. Briefly, the surfaces of the filters were coated with 15 μl ice-cold Matrigel (15 mg/ml protein; BD Tranduction) for 60 min at room temperature. Uniformity of the coating was checked by Coomassie blue staining and low-power microscopy observation. The lower chamber was filled with medium containing 10% serum. Fibronectin (16 μg/chamber) was added as the chemoattractant to the lower chamber. Cells (1 × 10^5 ^cells/well) were washed with 1 × PBS twice, re-suspended in 200 μl of serum-free medium and then transferred into the upper chamber. After 24 h of incubation, the filter was gently removed from the chamber, the cells on the upper surface were removed by wiping with a cotton swab, and cells that had invaded to the lower surface areas were fixed, stained with hematoxylin and eosin (H&E) and counted in 15 randomly selected fields on microscope (×100). Experiments were performed independently at least three times.

### Cell migration assay

Cell motility was assessed by scratch-wound assay as described previously [4]. The cells were seeded into 60-mm culture dishes at 2.5 × 10^5 ^cells and cultured in medium containing 10% FBS to nearly confluent cell monolayers, which were then carefully wounded by use of a 200-μl sterile pipette tip, and any cellular debris was removed by washing with PBS. The wound monolayers were then incubated in medium containing 10% FBS for 24 h and photographed under a light microscope (×200). The experiments were repeated in triplicate wells at least three times. The area of migrating cells was estimated by counting the number of pixels after the photographs had been converted to photoshop data (Adobe, San Jose, CA).

### Luciferase reporter assays

Cells at 50% confluence in 35-mm dishes were transfected by use of Superfect reagent. An amount of 1 μg of E-cadherin promoter gene construct (E-cadK1-*Luc*) or the E-box mutant construct and 1 μg of Id2 or its mutants were cotransfected. pRL-SV40 (Promega, Madison, WI) was used as an inner control (1 ng/well). Cell extracts were prepared 42 h after transfection, and the luciferase activity was measured by the Dual-Luciferase Reporter Assay System (Promega) as described previously [8]. All experiments were performed in triplicate and repeated three times.

### Statistical analysis

Data are expressed as mean ± S.E.M. Statistical differences were determined by use of the Chess software and Student's *t *test. *P *< 0.05 was considered statistically significant.

## Results

### Stable expression of Id2 and its mutants in MCF-7 and SKOV-3 cells

**Figure 1:**
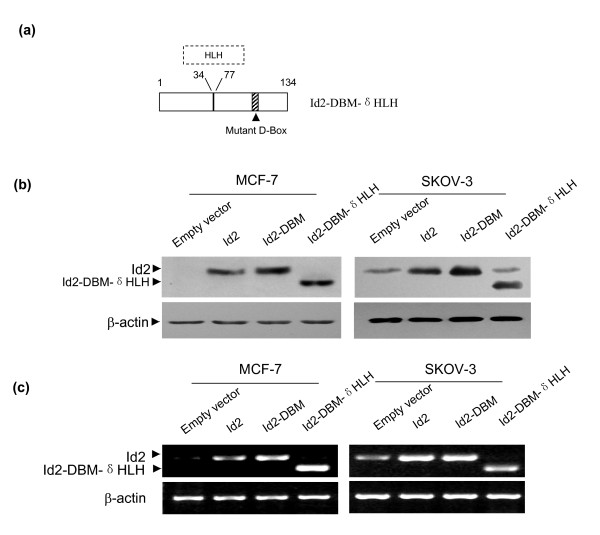
**(A) Schematic illustration of the Id2-DBM-δHLH construct**. In this construct, the HLH domain encoded by amino acid residues from 35 to 76 was deleted from Id2, and the key arginine and leucine residues within the D-box motif (RxxLxxxN) at residues 100 to 107 were changed to glycine and valine. **(B) Western blot analysis of Id2 and its mutants in MCF-7 and SKOV-3 cells**. Total proteins were extracted from cells transfected with the indicated plasmids, and then underwent SDS-PAGE electrophoresis and immunoblotting analysis using the indicated antibodies. β-actin was used as the loading control. **(C) RT-PCR analysis of Id2 and its mutants in MCF-7 and SKOV-3 cells**. Total RNA was extracted from cells transfected with the indicated plasmids and used for RT-PCR analysis of the mRNA expression of Id2 and its mutants. β-actin was the loading control. The results shown in B and C are representative of three independent experiments.

To investigate the effects of overexpression of Id2 and its mutants on the phenotypic traits of MCF-7 and SKOV-3 cells, the expression plasmids for Id2, Id2-DBM, and Id2-DBM-δHLH (schematic structure illustrated in Figure 1A) or the empty control vector were stably transfected into cells. After 10- to 14-day G418 screening, the drug-resistant clones appeared and were mixed for amplified culture. All of the parental cells were killed by G418 within this period. On western blot analysis, endogenous Id2 protein was not detected in MCF-7 cells and weakly detected in SKOV-3 cells, but ectopic transfection of Id2 markedly increased the expression of Id2 protein in both cell lines (Figure 1B). Ectopic transfection of D-box mutant Id2 further increased Id2 protein expression 2 to 3-fold that of transfection with the wild type Id2. A band corresponding to HLH-deleted Id2-DBM was detected using a rabbit anti-Id2 monoclonal antibody. Next, we further measured the mRNA levels of Id2 and its mutants in transfected cells by RT-PCR (Figure 1C) and found the mRNA expression of Id2 are consistent with protein levels in empty vector- and wild-type Id2-transfected cells. However, the mRNA level with Id2-DBM transfection did not differ from that with wild-type Id2 transfection in either MCF-7 or SKOV-3 cells. Id2-DBM-δHLH mRNA could be detected in cells, which confirms the effective expression of the HLH-deleted Id2. Collectively, these data indicated that the ectopic Id2 and its mutants can effectively be expressed in MCF-7 and SKOV-3 cells and with the D-box mutation transfection, Id2 protein could accumulate to a much higher level than with the wild type.

### Overexpression of Id2 and its mutants does not promote proliferation of MCF-7 or SKOV-3 cells

**Figure 2:**
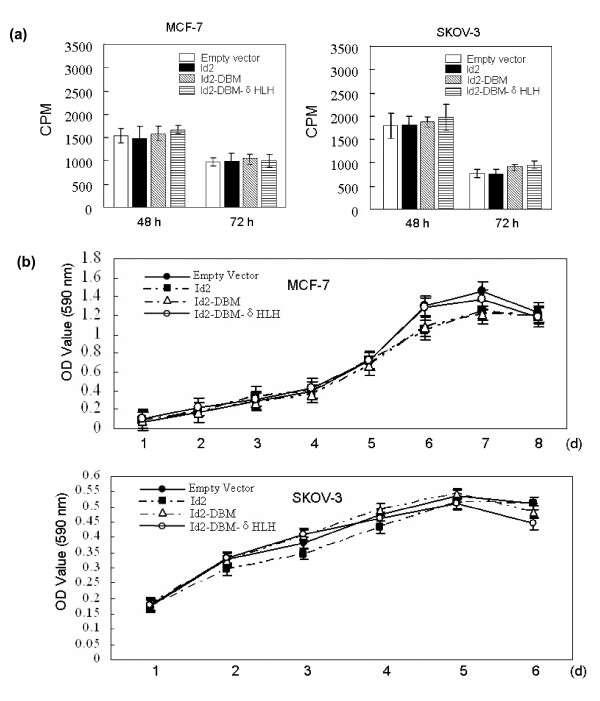
**(A) Effect of overexpression of Id2 or its mutants in MCF-7 and SKOV-3 cells on [^3^H] thymidine incorporation into cellular DNA**. The indicated cells transfected with the indicated plasmids were seeded in 24-well plates in culture media with 10% FBS (1 × 10^4^/well). At the indicated times, [^3^H]-thymidine (2 μCi) was added to fresh medium and incubated for 1 h at 37°C prior to scintillation counting. Experiments at each time point were performed in triplicate, and the graph is representative of three independent experiments. **(B) Effect of overexpression of Id2 or its mutants on proliferation rate of MCF-7 and SKOV-3 cells**. The indicated cells transfected with the indicated plasmids were cultured in triplicate, and MTT assay was performed at the indicated times. Absorbance was examined at 590 nm by use of a microplate reader. Data are mean ± SEM for at least three separate determinations. CPM = counts per million; OD = optical density.

Overwhelming evidence favors the view that Id proteins are essential proliferative factors for a large variety of cell types [9,10]. To determine whether overexpression of Id2 and its mutants promotes proliferation of MCF-7 and SKOV-3 cells, we performed [^3^H]-thymidine incorporation and MTT assays and revealed that transfection of Id2 and its mutants did not significantly alter proliferation of either cell line (Figure 2A and 2B).

### Overexpression of Id2 and its mutants promotes the in vitro invasion and motility of MCF-7 and SKOV-3 cells

**Figure 3:**
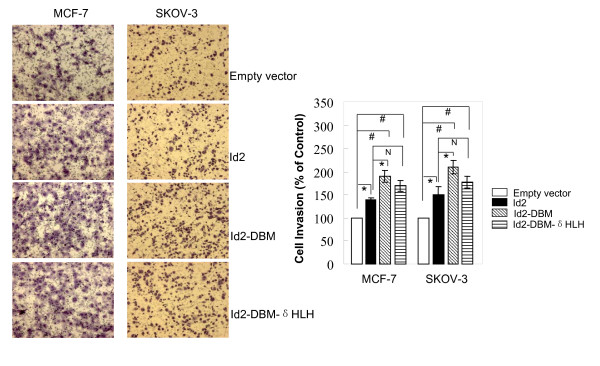
**Effect of overexpression of Id2 or its mutants on the *in vitro *invasion of MCF-7 and SKOV-3 cells**. The left panel is representative pictures of Transwell assays, performed for the indicated cells as described in "*Methods*". Each experiment was performed in triplicate and was repeated three times. Data are expressed as the percentage of the control cells (mean ± SEM, the bottom panel). (*P < 0.05; # *P *<*0.01*; N *P *> *0.05*).

To examine the effects of Id2 and its mutants on the invasive capacity of MCF-7 and SKOV-3 cells, we performed a modified Boyden chamber assay to determine the ability of cells to invade through biological matrices *in vitro*. The relevance of this assay for other invasive assays and for *in vivo *malignancy has been documented extensively [25]. Both MCF-7 and SKOV-3 are poorly invasive cell lines, but their invasion potential is moderately increased in the presence of fibronectin as the chemoattractant. The invasion of cells with ectopic transfection of wild-type Id2 or its two mutants was higher than that of the control cells (Figure 3, left panel). In contrast to mock transfection, Id2 transfection produced a 40% and 50% increase of the invasiveness of MCF-7 and SKOV-3 cells, respectively (Figure 3, right panel). Unexpectedly, such invasion was augmented to a higher level by Id2-DBM or Id2-DBM-δHLH transfection in both cell lines with an increase in invasiveness of 90% and 70% for MCF-7 cells and 110% and 78% for SKOV-3 cells with Id2-DBM and Id2-DBM-δHLH transfection, respectively (Figure 3). These results pointed to a linear link between the invasive capacity of MCF-7 and SKOV-3 cells and Id2 expression level and also indicate that this functional link did not depend on the presence of HLH domain of Id2. Overexpression of the HLH-deleted Id2 induced a significant increase in invasiveness of MCF-7 and SKOV-3 cells.

**Figure 4:**
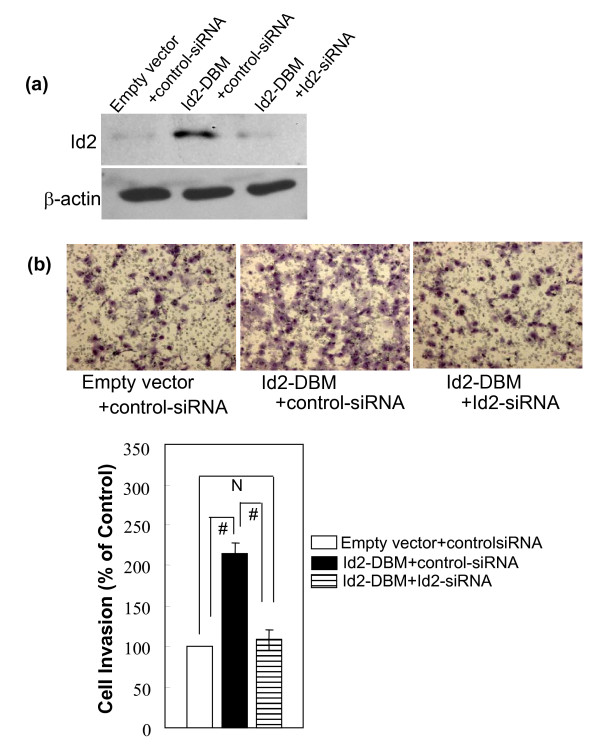
**(A) Effect of Id2-specific siRNA transfection on the expression of the exogenous Id2-DBM protein in MCF-7 cells**. The indicated siRNA oligonucleotides were transfected into the indicated MCF-7 cells. 48 h after transfection, total proteins were extracted and subjected to immunoblotting analysis with Id2-specific antibody. β-actin was used as the loading control. **(B) Effect of knock down of exogenous Id2-DBM by RNA interference on in vitro invasion of MCF-7 cells**. The indicated siRNA oligonucleotides were transfected as described in (A). 48 h after transfection, MCF-7 cells were subjected to performing Transwell experiments. The top panel shows representative pictures of Transwell assays, as described in "*Methods"*. Each data was performed in triplicate and was repeated three times. Data are expressed as the percentage of the control cells (mean ± SEM, the bottom panel). (# *P *<*0.01*; N *P *> *0.05*).

To further address the promotion role of Id2 overexpression on cell invasiveness, Id2-specific siRNA or control-siRNA oligonucleotides was transiently transfected into MCF-7 cells stably expressing Id2-DBM or pcDNA3.1 empty vector. Forty-eight hours later, cells were harvested and subjected to western blot analysis and Transwell experiments. The expression of ectopic Id2-DBM protein was largely inhibited by the Id2-specific siRNA but not by control-siRNA (Figure 4A). Knock down of exogenous Id2-DBM in MCF-7 cells by Id2-specific siRNA cotransfection reduced the cell invasiveness enhanced by Id2-DBM to that of the control cells (Figure 4B).

**Figure 5:**
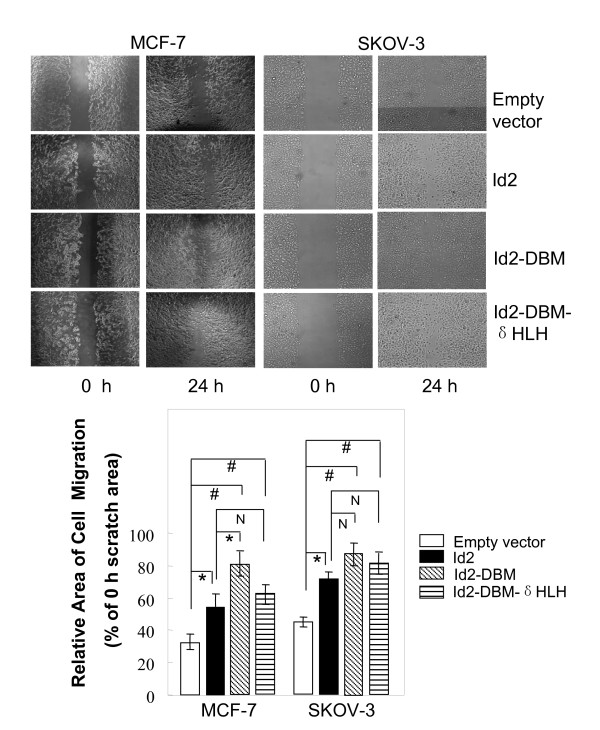
**Effect of overexpression of Id2 or its mutants on the migratory capacity of MCF-7 and SKOV-3 cells**. Migratory behaviors of Id2 or its mutant transfectants were determined by scratch-wound assay (top panel) and quantified as described in "*Methods" *(bottom panel). The scratch area at 0 h was arbitrarily assigned as 100%. (*P < 0.05; # *P *<*0.01*; N *P *> *0.05*).

To examine whether the invasion potential of MCF-7 and SKOV-3 cells promoted by Id2 and its mutants was associated by their increase in cell motility, we analyzed the effects of Id2 and its mutants on the migration capability of cells by observing the effects of scratch wounding confluent monolayers of cells transfected with Id2 or its mutants. Overexpression of the wild-type Id2 or its mutants markedly promoted the flattening and spreading of both cell lines along the edges of the wound as compared with the control (Figure 5).

### Id2 mutants but not the wild-type Id2 inhibit the expression and transcriptional activity of *E-cadherin *gene in MCF-7 and SKOV-3 cells

**Figure 6:**
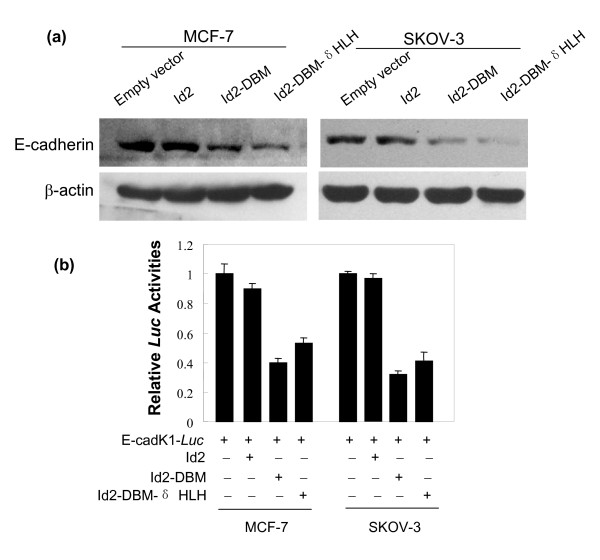
**(A) Effect of overexpression of Id2 or its mutants on E-cadherin protein expression**. Total proteins were extracted from the indicated cells for analysis of E-cadherin protein by western blot. **(B) Effect of overexpression of Id2 or its mutants on the transactivation of *E-cadherin *gene promoter**. The indicated cells were transiently cotransfected with the indicated plasmids for luciferase assay as described in "*Methods*". Data are expressed as mean ± SEM for at least three separate determinations.

E-cadherin (encoded by the *CAD1 *gene in humans) is a cell-surface glycoprotein involved in calcium-dependent cell-cell adhesion [26]. The increased invasion and migratory capacity of epithelial tumor cells are always associated with reduced level of E-cadherin [27-29]. To ascertain whether Id2-induced increase of *in vitro *invasiveness and migration of MCF-7 and SKOV-3 cells is accompanied by changes in E-cadherin, we analyzed the protein expression of *E-cadherin *by western blot assay. Transfection of the wild-type Id2 did not significantly change the expression of E-cadherin protein in either cell line (Figure 6A); however, transfection with Id2-DBM or Id2-DBM-δHLH caused a 2- to 5-fold decrease of E-cadherin protein expression as compared with the mock transfection in both cell lines. Next, to determine whether the Id2 mutants are repressors of transcriptional activity of *E-cadherin *gene, the promoter construct of *E-cadherin*, Id2 or either of its mutants were cotransfected into MCF-7 and SKOV-3 cells. Since the proximal *E-cadherin *promoter was shown to mainly confer the transcriptional inactivation of *E-cadherin *in some cancer cell lines [30-33], we used the most proximal *E-cadherin *human promoter construct (EcadK1-*Luc*, -108 to +125), which contained three E-box elements. E-cadK1-*Luc *activities were efficiently repressed by Id2-DBM and Id2-DBM-δHLH (2- to 2.5- fold) but not by the wild-type Id2 (Figure 6B). Thus, excessive accumulation of Id2 by abolishing its degradation in MCF-7 and SKOV-3 cells represses the expression of *E-cadherin *gene by inhibiting its transactivation. As well, this repression is not dependent on the HLH domain of Id2.

**Figure 7:**
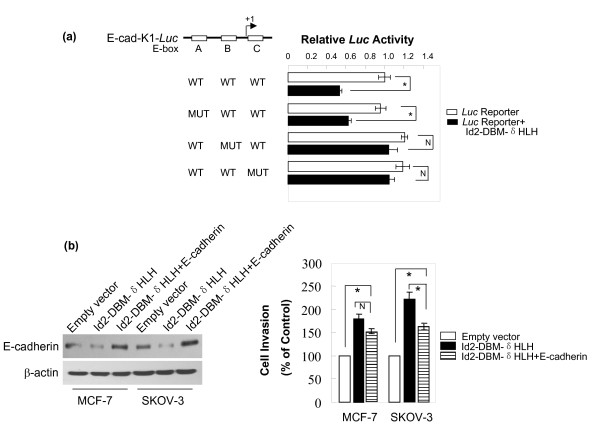
**(A) Effect of E-box mutagenesis on Id2-DBM-δHLH repression of *E-cadherin *promoter reporter activity**. MCF-7 cells were transiently cotransfected with the indicated plasmids for luciferase assay as described in "*Methods*". Data are expressed as mean ± SEM for at least three separate determinations. **(B) Effect of ectopic E-cadherin expression on Id2-DBM-δHLH-induced cell invasion potential**. E-cadherin expression vector or the corresponding empty vector was transiently transfected into Id2-DBM-δHLH stably expressed or wild-type MCF-7 and SKOV-3 cells for immunoblotting analysis with the indicated antibodies (left panel) and Transwell assays (right panel). Data are expressed as the percentage of the control cells (mean ± SEM). (# *P *<*0.01*; N *P *> *0.05*).

To identify whether the E-box contained within the proximal region of *E-cadherin *promoter is responsive to HLH-deleted Id2-DBM, we used E-box A, B or C mutant constructs and Id2-DBM-δHLH to cotransfect MCF-7 cells for luciferase assays. Mutation of E-box B or E-box C but not A significantly attenuated the repression of the reporter gene activity by Id2-DBM-δHLH (Figure 7A), which suggests that the E-box-binding transcription repressors are involved in HLH domain-independent Id2 repression of *E-cadherin *transactivation.

To test whether the induction of the cells' invasion capacity by Id2-DBM-δHLH overexpression is attributed to the down-regulation of E-cadherin, the E-cadherin expression vector or pcDNA3.1 empty plasmid was transiently transfected into Id2-DBM-δHLH-overexpressing MCF-7 and SKOV-3 cells. Immunoblotting analysis showed E-cadherin protein level augmented in E-cadherin-transfected cells (Figure 7B, left panel,). Although the invasiveness of transfectants with Id2-DBM-δHLH plus E-cadherin was significantly higher than that of the empty-vector transfectants, the ectopic expression of E-cadherin in both MCF-7 and SKOV-3 cells could partially blunt the invasion potential induced by Id2-DBM-δHLH (Figure 7B, right panel,). Thus, down-regulation of E-cadherin expression is responsible only in part for the HLH domain-independent Id2-enhanced invasion, and over-accumulated Id2 could induce other unknown signaling through its non-HLH region to promote cell invasion.

## Discussion

Tumor progression is the evolution of already tumorigenic cells towards increasing malignancy [1,2]. During tumor progression, further aberrant molecular events may occur in a specialized subset of low invasive cancer cells and ultimately promote these cells to acquire the ability of highly invasive growth [3]. Identification of the molecules that predispose tumor cells to a more invasive phenotype is helpful for understanding the tumor metastasis process as well as providing potential therapeutic targets for tumor progression. In this study, we manipulated Id2 expression in poorly invasive MCF-7 and SKOV-3 cancer cells and determined the effects on cell proliferation, *in vitro *invasion and migration. Id2 could facilitate the invasive and migratory capabilities of MCF-7 and SKOV-3 cells in a dose-dependent manner. Importantly, structure and function analyses revealed that the HLH domain of Id2 is not required for its pro-invasive function. These observations suggest that the aberrant accumulation of Id2 in some specific non-aggressive epithelial tumor cells may be sufficient to convert them into more invasive cells.

Elevated levels of Id2 expression have been reported in carcinomas of breast, ovary, colon and prostate, in neural tumors, melanoma, Ewing's sarcoma and in hematological malignancies [13-15]. In some cases, high levels of Id2 expression are associated with disease severity and poor prognosis. However, significant contradictions exist in various types of human tumors. In addition, cell-based experiments indicated that Id2 has diverse and complex biological effects depending on cell lineage, differentiation state, and other contextual considerations [24]. For example, down-regulation of Id2 expression in highly metastatic PC-3 human prostate cancer cells reduced their growth potential and invasiveness, which indicates the pro-proliferation and pro-invasion roles of Id2 in some epithelial cancer cells [34]. Conversely, ectopic expression of Id2 in MDA-MB-231 and MDA-MB-436 human breast cancer cells did not significantly affect cell growth but markedly reduced the cells' invasive capacity [23,35]. These controversial functional consequences of Id2 on tumor growth and invasion suggest the diverse nature of Id2 target signaling pathways in different cell contexts. Therefore, careful evaluation is required to unambiguously identify the tumor cell types or subtypes that may use Id2 to control their different phenotypes. Although MCF-7 is also a human breast cancer cell line, its genetic background and phenotypic characteristics differ greatly in MDA-MB-231 and MDA-MB-436 cells. Generally, the MCF-7 cell line is representative of ERα-positive and non-aggressive human breast cancers; however, MDA-MB-231 and MDA-MB-436 are representative of ERα-negative breast cancer cells. In addition, SKOV-3 is an ovarian carcinoma cell line with ERα-positive and non-aggressive phenotypes. In this study, overexpression of Id2 or either of its two mutants increased the invasive capacity of MCF-7 and SKOV-3 cells but did not alter the proliferation of either of these two cell lines. The functional role of Id2 in the cell invasion phenotype in MCF-7 and SKOV-3 cells is completely contrary to that previously observed in MDA-MB-231 and MDA-MB-436 cells. Considering the expression of ERα in MCF-7 and SKOV-3 cells, we postulated that the aberrant Id2 expression may play an important role in converting ERα-positive epithelial tumor cells into highly invasive cells. Although a high expression of Id2 in primary breast cancer cells was reported to confer favorable clinical outcome [35], our findings suggest that the analysis of Id2 expression in combination with ERα status may be better for prognostic reevaluation of breast cancer.

Our important finding is that the HLH domain of Id2 is not required for the pro-invasive activity of the protein and that the level of expression of an invasion-suppressing molecule, E-cadherin, is down-regulated by only the degradation-resistant Id2 form. These observations raise the possibility that different expression levels of Id2 can influence different gene expression through a heretofore unknown transcriptional activity of its non-HLH region. Consistent with this is our observation that the transactivation of E-cadherin promoter is significantly suppressed by the degradation-resistant full-length and HLH-deleted Id2 forms but not the wild-type form. Although MCF-7 and SKOV-3 cells are highly sensitive to changes in the levels of E-cadherin and will undergo enhanced invasion if E-cadherin is down-regulated, supplementation of E-cadherin in cells does not completely antagonize the invasion potential induced by Id2-DBM-δHLH. Overexpression of the wild-type Id2 in MCF-7 and SKOV-3 cells did not reduce E-cadherin expression but did indeed promote the invasion and migration of both cell lines. So, different mechanisms are involved in the invasion potential induced by different Id2 expression levels in cells.

Previous studies have linked Id and E-cadherin expression in some specific cell types. Id proteins have been shown to activate E-cadherin in normal epithelial cells by inhibiting E2A protein, which represses the most proximal E-cadherin promoter in these cells through interaction with the E-box elements [36,37]. However, apparent contradiction exists in some specific cell types. For example, in uveal melanoma Mel202 and Mel290 cells, Id2 suppressed E-cadherin expression through inhibiting the transactivation of its proximal promoter by an unknown regulatory mechanism [33]. In this study, Id2 suppressed E-cadherin expression in MCF-7 and SKOV-3 cells through a mechanism independent of its dimerization to bHLH factors when Id2 was accumulated to a high level in cells.

E-cadherin is often positive in ERα-positive cancer cells and often negative in ERα-negative tumor cells (such as MCF-7 versus MDA-MB-231 cells), and loss of E-cadherin may result in the more aggressive growth of ERα-positive cells by increasing the probability of invasion and metastasis [26]. Epithelial cancers arise within an epithelium where cells are constrained by E-cadherin-mediated cell-cell interactions. Hence, in the early stages of these cancers, E-cadherin must be down-regulated to escape the local epithelial environment and invade local structures. In this paradigm, the aberrant accumulation of Id2 and the subsequent E-cadherin down-regulation as we described should provide a selective growth advantage in the tumor microenvironment by increasing the probability of invasion and metastasis.

## Conclusion

In summary, in our analyses of the expression of Id2 and its mutants in ERα-positive MCF-7 and SKOV-3 cells, aberrant accumulation of Id2 in certain ERα-positive epithelial tumor cells indeed increased the cells' invasive potential through a novel mechanism independent of dimerization to bHLH factors and E-cadherin partially contributes to Id2-induced cell invasion in an HLH domain-independent manner when Id2 is accumulated to a high level in some specific cell types.

## Competing interests

The authors declare that they have no competing interests.

## Authors' contributions

WH participated in the design of the study, performed the experimental data acquisition, performed data analyses and interpretation and drafted the manuscript. YM participated in the design of the study and performed some of the experiments. CG, ZW, YZ and YS performed partial experiments and participated in data analyses and interpretation. XF participated in data analysis and discussion and critically revised the manuscript. All authors read and approved the final manuscript.

## Pre-publication history

The pre-publication history for this paper can be accessed here:

http://www.biomedcentral.com/1471-2407/9/75/prepub
